# Algicidal characteristics of novel algicidal compounds, cyclic lipopeptide surfactins from *Bacillus tequilensi*s strain D8, in eliminating *Heterosigma akashiwo* blooms

**DOI:** 10.3389/fmicb.2022.1066747

**Published:** 2022-11-30

**Authors:** Xueping Shao, Wanxin Xie, Yiling Liang, Guiying Luo, Ling Li, Wei Zheng, Qingyan Xu, Hong Xu

**Affiliations:** ^1^State Key Laboratory of Cellular Stress Biology, School of Life Sciences, Xiamen University, Xiamen, Fujian, China; ^2^Key Laboratory of the Ministry of Education for Coastal and Wetland Ecosystems, Xiamen University, Xiamen, Fujian, China

**Keywords:** *Heterosigma akashiwo*, surfactin, algicidal characteristics, esterase activity, photosynthetic function, harmful algal bloom

## Abstract

*Heterosigma akashiwo* blooms have caused severe damage to marine ecosystems, the aquaculture industry and human health worldwide. In this study, *Bacillus tequilensis* D8 isolated from an *H. akashiwo* bloom area was found to exert high algicidal activity *via* extracellular metabolite production. This activity remained stable after exposure to different temperatures and light intensities. Scanning electron microscopy observation and fluorescein diacetate staining indicated that the algicidal substances rapidly destroyed algal plasma membranes and decreased esterase activity. Significant decreases in the maximum photochemical quantum yield and relative electron transfer rate were observed, which indicated photosynthetic membrane destruction. Subsequently, the algicidal compounds were separated and purified by high-performance liquid chromatography and identified as three surfactin homologues by interpreting high-resolution electrospray ionization mass spectrometry and nuclear magnetic resonance spectroscopy data. Among these, surfactin-C13 and surfactin-C14 exhibited strong algicidal activity against three HAB-causing species, namely, *H. akashiwo, Skeletonema costatum*, and *Prorocentrum donghaiense*, with 24 h-LC_50_ values of 1.2–5.31 μg/ml. Surfactin-C15 showed strong algicidal activity against *S. costatum* and weak algicidal activity against *H. akashiwo* but little activity against *P. donghaiense*. The present study illuminates the algicidal characteristics and mechanisms of action of surfactins on *H. akashiwo* and their potential applicability in controlling harmful algal blooms.

## Introduction

During the past three decades, large-scale and high-frequency outbreaks of harmful algal blooms (HABs) have endangered marine ecosystems and human health (Brooks et al., 2016; Grattan et al., 2016). *Heterosigma akashiwo*, a global source of HABs, causes discoloration of water bodies and the death of fish and shellfish (Ling and Trick, 2010; Siano et al., 2020), severely damaging tourism and aquaculture development and hindering the development of coastal economies (Haigh and Esenkulova, 2014). Therefore, it is imperative to find a feasible method for controlling *H. akashiwo* blooms.

The current strategies for controlling HABs mainly include physical [e.g., flocculation (Pang et al., 2013)], chemical [e.g., copper sulfate (Gallardo-Rodríguez et al., 2019)], and biological [e.g., allelopathy (Hua et al., 2018)] approaches. Biological methods are more suitable for HAB prevention and control due to their high efficiency and environmental friendliness. Microbial control, which kills algal cells directly or indirectly, is the most efficient biological method. In direct microbial control, microorganisms directly attack the algal cell membrane or cell wall to cause algal cell lysis (Caiola and Pellegrini, 2004; Chen et al., 2017). In indirect microbial control, algal cell death occurs *via* the production of extracellular substances (Sun et al., 2018). Various algicidal substances produced by bacteria, including alkaloids, pigments, amino acids, cyclic peptides and peptide derivates, glycolipids, lipopeptides and fatty acids, have been reported (Hirao et al., 2012; Zhang et al., 2020; Quan et al., 2021). However, only a few metabolites with high algicidal activity against *H. akashiwo* have been identified. Rhamnolipids produced at low concentrations (4 μg/mL) by *Pseudomonas aeruginosa* have been found to induce algae cell lysis rapidly by anchoring onto the phospholipid bilayer of *H. akashiwo* (Wang et al., 2005). Prodigiosin derived from *Hahella* caused the excessive production of reactive oxygen species (ROS), changed the transcription levels of photosynthetic genes (*psbA, psbD*) and respiration-related genes (*cob, cox1*) and damaged the photosynthetic system of *H. akashiwo* (Zhang et al., 2020). Similarly, Nω-acetylhistamine secreted by *Bacillus* sp. strain B1 caused oxidative stress and malondialdehyde accumulation, which significantly inhibited *H. akashiwo* growth. Nω-acetylhistamine (40 μg/mL) mainly induced algal cell death and markedly reduced the toxicity of *H. akashiwo* (Zhu et al., 2021). Ortho-tyrosine produced by *Bacillus* mainly damaged the membrane structure of *H. akashiwo*, resulting in cell lysis. In addition, 300 μg/mL o-tyrosine significantly inhibited the photosynthetic system, reduced the mitochondrial membrane potential, and increased the membrane permeability of algal cells. In contrast, urocanic acid had little effect on membrane permeability and the photosynthetic system but decreased the mitochondrial membrane potential, thereby inhibiting *H. akashiwo* growth and reproduction (Quan et al., 2021).

Surfactin, a cyclic lipopeptide produced by various strains of the *Bacillus* genus, comprises a heptapeptide interlinked with β-hydroxy fatty acid chains with lengths of 12–16 carbon atoms, forming a cyclic lactone ring structure (Seydlová and Svobodová, 2008). Surfactins are actually considered a family of lipopeptides, sharing common structural characteristics with great structural diversity due to the type of amino acids in the heptapeptide ring and the length of the lipidic chain. Different surfactin isoforms coexist in cells as a mixture of seven peptide variants with different aliphatic chain lengths (Théatre et al., 2021). Due to its hydrophilic peptide moiety and lipophilic fatty acid chain moiety, surfactin is an amphipathic biosurfactant and exhibits potent surfactant properties, reducing the surface tension of water from 72 to 27 mN/m at concentrations as low as 10 μM (Datta et al., 2020). Due to their excellent ability to attack the cell membrane and improve the bioavailability of hydrophobic organics (Dewidar and Sorial, 2021), surfactins are often used in antibacterial (Johnson et al., 2021), antimycoplasmal (Wang et al., 2020b), antiviral (Yuan et al., 2018), anticancer (Wu et al., 2017), and oil recovery and degradation applications (Yang et al., 2020; Meena et al., 2021). However, surfactins have never been applied to kill algae and control HABs. Chemical surfactants and clay have been combined and applied for HAB control (Wang et al., 2017). Compared with chemical surfactants, biosurfactants have the advantages of being biodegradable, not causing secondary pollution, and being non-toxic or having low-toxicity (Roy, 2017). Therefore, surfactins, as biosurfactants, may be suitable for controlling HABs, decreasing their occurrence or even eliminating them.

In this study, we isolated the D8 algicidal strain from a Xiamen coastal environment during a *H. akashiwo* bloom and identified it as *Bacillus tequilensis* based on 16S rDNA sequencing and morphological observation. The D8 strain exhibited the potential for bloom control *via* the production and secretion of algicidal compounds. We first explored the algicidal properties and mechanisms of these algicidal compounds. Then, we separated, purified and identified the algicidal components as three surfactin homologues through high-performance liquid chromatography (HPLC), high-resolution electrospray ionization mass spectrometry (LC-MS/MS), and nuclear magnetic resonance (NMR) spectroscopy. Finally, we measured the half-lethal concentrations (LC_50_) of each surfactin for three HAB-causing species, namely, *H. akashiwo, Skeletonema costatum*, and *Prorocentrum donghaiense*. This study reports the algicidal activity of these surfactins and lays a strong foundation for the development of an efficient algicide for the control of HABs caused by *H. akashiwo, S. costatum*, and *P. donghaiense*.

## Materials and methods

### Microalgae, algicidal bacteria, and culture medium

*H. akashiwo, S. costatum*, and *P. donghaiense* were obtained from the Algal Species Management Center, School of Ocean and Earth, Xiamen University (China). The algal cultures were grown in f/2 medium (Guillard, 1975) at 20∼22°C under a 12:12 h light: dark cycle with the light intensity maintained at 50 μmol photons m^–2^ s^–1^.

The algicidal bacterium D8 was originally isolated from water samples that were collected from Xiamen’s first wharf in Fujian Province, China, during a *H. akashiwo* bloom. It was grown in STA medium (Hetharua et al., 2018) at 28∼30°C with a shaking speed of 150 rpm. Isolation and purification of strain D8 were carried out according to a previous study (Zhang et al., 2018).

### Identification of the bacterial strain

Bacterial D8 was characterized by its 16S rRNA gene. The 16S rRNA gene was amplified by PCR using primers 27F and 1492R. The PCR product of the 16S rRNA gene was purified using the TaKaRa MiniBEST DNA purification kit (TaKaRa Bio Inc., Dalian, China) and then sequenced. The sequence was submitted to EzBioCloud^1^ for sequence alignment. Phylogenetic analysis was performed using MEGA version 5.0 software and neighbor-joining analysis. The cell morphology was observed by SEM (JSM-6390, JEOL Co., Tokyo, Japan) and TEM (JEM-2100HC, JEOL Co., Tokyo, Japan).

### Algicidal activity and mode assays

To investigate the algicidal mode, the cell-free supernatant of the D8 culture (20 ml) was collected by centrifugation at 6,000 rpm for 10 min and then filtered through a 0.22 μm membrane. The cell pellet was resuspended in 20 ml of sterile f/2 medium for the subsequent analysis of direct algicidal activity. The different fractions of the D8 culture were added to the algal cultures (10 ml) at a concentration of 5% (v/v). After 48 h of treatment, the algicidal rate of each fraction was calculated by the following formula:


$$Algicidal rate\left(\%\right)=\left[\left({\text{N}}_{0}-{\text{N}}_{t}\right)/{\text{N}}_{0}\right]\times \text{1}00\%$$

where N_0_ represents the cell number of the algal cultures measured immediately after treatment, and N_*t*_ represents the cell number of the algal cultures at different treatment times.

### Acquisition and algicidal activity of D8 crude extracts

Strain D8 was cultured in 500 ml of STA medium for 72 h, and then the supernatant was obtained by centrifugation at 8,000 rpm for 10 min. The pH of the D8 supernatant was adjusted to 2.0 with 6 M HCl, and the solution was kept at 4°C overnight. Then, the precipitate was collected by centrifugation and extracted once with 20 ml of chloroform:methanol (2:1) and twice with 20 ml methanol. The methanol was removed using a rotary evaporator to obtain the D8 crude extract. The D8 crude extract (20 mg) was dissolved in 1 ml of DMSO for further analysis. To assess the algicidal activity of the D8 crude extract, 2.5, 5, 10, 15, or 20 μg/mL D8 crude extract was inoculated into *H. akashiwo* cultures, and the algicidal activity was calculated according to the above formula.

### Chlorophyll *a* content, Fv/Fm, and relative electron transport rate measurements

To assay the photosynthetic response of *H. akashiwo* exposed to the D8 crude extract, the Chl *a* content, carotenoid content, maximum quantum yield of photosystem (PS) II (Fv/Fm) and relative electron transport rate (rETR) of the algal cells were measured after treatment with D8 crude extract. The algal cultures (100 ml) were treated with 2.5, 5, or 10 μg/ml of D8 crude extract, and 10 ml samples were collected after 3, 6, 12, and 24 h of exposure. The Chl *a* and carotenoids in the collected samples were extracted with 2 ml of 95% alcohol. The pigment levels were calculated by measuring the absorbance at 665, 645, and 470 nm, and applying the following equations:


$$\text{Chlorophyll}\;a\text{(mg}/\text{L})=\text{12}.\text{7}\times {\text{A}}_{\text{665}}-\text{2}.\text{69}\times {\text{A}}_{645}(Marr et al.,1995)$$

$$\text{Carotinoid}\;\left(\text{mg/l}\right)=\left(\text{1}000\times {\text{A}}_{\text{47}0}-\text{2}.0\text{5}\times {\text{C}}_{Chlorophyll\;a}\right)/245(Zuo et al.,20\text{2}0)$$

where A_665_, A_645_, and A_470_ represent absorbance values at wavelengths of 665, 645, and 470 nm, respectively, and C_*Chlorophyll*_ *_*a*_* represents the Chl *a* content.

The Fv/Fm and rETR values of treated samples (2 ml) were measured using a multiple excitation-wavelength modulated chlorophyll fluorometer (Multi-Colour-PAM, Walz, Oberschwaben, Germany) according to our previous study (Zhang et al., 2018).

### Cell viability measurements

Fluorescein diacetate (FDA) was used to assess the viability of *H. akashiwo*. FDA is a non-polar molecule and can freely enter living cells, where it is digested by esterase to produce fluorescein. Fluorescein is a polar molecule and cannot penetrate the cell membrane. Therefore, digested fluorescein accumulates in living cells and emits green fluorescence. The ratio of fluorescein-positive cells to total cells indicates cell viability. To determine the effect of D8 crude extract on cell viability, algal cells were treated with D8 crude extract at final concentrations of 2.5, 5, and 10 μg/ml. After treatment for 5, 30, 60, and 180 min, the algal cells were collected by centrifugation and stained with 20 μg/mL FDA (F809625, Beijing Huawei Ruike Chemical Co., Ltd, Peking, China) for 5 min in the dark at room temperature. Fluorescence was analyzed on a flow cytometer (BD LSRFortessa, Becton, Dickinson and Company, New Jersey, USA) using 488 nm excitation and a 525 nm bandpass filter for digested fluorescein detection. For each sample, approximately 5,000 cells were analyzed. Fluorescence images were collected by a fluorescence microscope (Leica DM4B, Lecia, Wetzlar, Germany).

### Morphological observation using scanning electron microscopy

Algal cells treated with 5 μg/ml D8 crude extract for 0, 0.5, 3, 6, 12, and 24 h were separately collected by centrifugation at 1,000 rpm for 5 min. The collected cells were fixed with 2.5% (v/v) glutaraldehyde overnight at 4°C and then washed once with f/2 medium. The fixed samples were observed using SEM (SUPRA 55 SAPPHIRE, Zeiss Optical Instruments Inc., Jena, Germany) according to our previous work (Zhang et al., 2018).

### Purification and identification of algicidal components

D8 crude extracts were further separated and purified by chromatographic methods using an octadecylsilyl (ODS) column (particle size 50 μm, Φ3.0 × 30 cm) with a loading volume of 2 ml (100 mg/ml D8 crude extract of methanol) at room temperature. Acetonitrile (mobile phase A) and 0.05% TFA/water (v/v) (mobile phase B) were used as the solvent system. The column was first washed with the initial mobile phase (80% A + 20% B) at a flow rate of 3 ml/min, and 25 ml aliquots were collected in test tubes. After filling the 8th tube, 100% A was used to elute the algicidal components. The eluate signal was detected using a spectrophotometer at a wavelength of 205 nm. Algicidal components were characterized by an analytical high-performance liquid chromatograph (Waters 2545, Waters Corporation, Milford, MA, USA) equipped with a reversed-phase HPLC column (Sunfire C18, Waters Corporation, USA, Φ4.6 mm × 50 mm, particle size 5 μm) at a flow rate of 1 ml/min.

Further purification and preparation were performed using a SilGreen C18 column (GH0525010C18A, Beijing Green Baicao Technology Development Co. Ltd, Peking, China, Φ10 mm × 100 mm, particle size 5 μm) at a flow rate of 4.5 ml/min. The eluate was monitored at 205 nm by preparative liquid chromatography (LC-20AP, Shimadzu, Kyoto, Japan). The mobile phase was 85% acetonitrile (0.05% TFA), and an isometric elution method was used. The target fraction was collected and evaporated under vacuum by a rotary evaporator (EYELA OSB-2100, Tokyo Rikakikai Co., Ltd, Japan) at 45°C.

### High-resolution electrospray ionization mass spectrometry (LC-MS/MS) analysis

Purified compounds were analyzed using a high-resolution LC-MS/MS instrument (Q-Exactive, Thermo Fisher Scientific Inc., Waltham, USA). LC–MS was conducted using an Orbitrap MS instrument equipped with an electrospray ionization (ESI) source. The mass spectrometer was operated in positive ion mode using ESI under the following conditions: a spray voltage of 3800 V and a capillary temperature of 320°C.

### Nuclear magnetic resonance spectroscopy

One-dimensional (^1^H-NMR, ^13^C-NMR) spectrograms of purified compounds were recorded using an NMR spectrometer (Bruker AV600, Bruker, Zurich, Switzerland) at 600 and 150 MHz for ^1^H and ^13^C NMR, respectively, with DMSO-d6 (δ_*H*_ 2.50 and δ_*C*_ 39.5 ppm) as the solvent. ^1^H-NMR spectra were obtained at 600 MHz with 40 scans, a frequency resolution of 0.09 Hz, and a relaxation delay time of 1 s using a spectral width of 12019.2 Hz. ^13^C-NMR spectra were obtained at 150 MHz with 1500 scans, a frequency resolution of 0.55 Hz and a relaxation delay time of 2 s using a spectral width of 36057.7 Hz.

### Algicidal activities of three purified compounds

To assess the algicidal activity of purified components, various concentrations (0.5, 1, 2, 3, 4, 5, 10, 15 20, 30, or 60 μg/ml) of each component were inoculated into *H. akashiwo, S. costatum*, and *P. donghaiense* cultures, respectively. The cell density of algal cultures was assessed and recorded using a Countstar analyzer (ALIT, Shanghai, China) every 12 h for 48 h after the treatment with each component. The algicidal activity was calculated according to the above formula. Treatments with the same volume of DMSO served as the control. All treatments were performed in triplicate.

### Statistical analyses

All of the experimental data were collected in triplicate and are presented as the means ± standard deviations. The statistical significance of the difference in parameters between the control and treatment groups was analyzed using GraphPad Prism 8.0 with two-way ANOVA, for which *P* < 0.05 was considered to indicate statistical significance. The LC_50_ values were calculated by probit analysis in SPSS.

## Results

### Algicidal activities and features of strain D8

**FIGURE 1:**
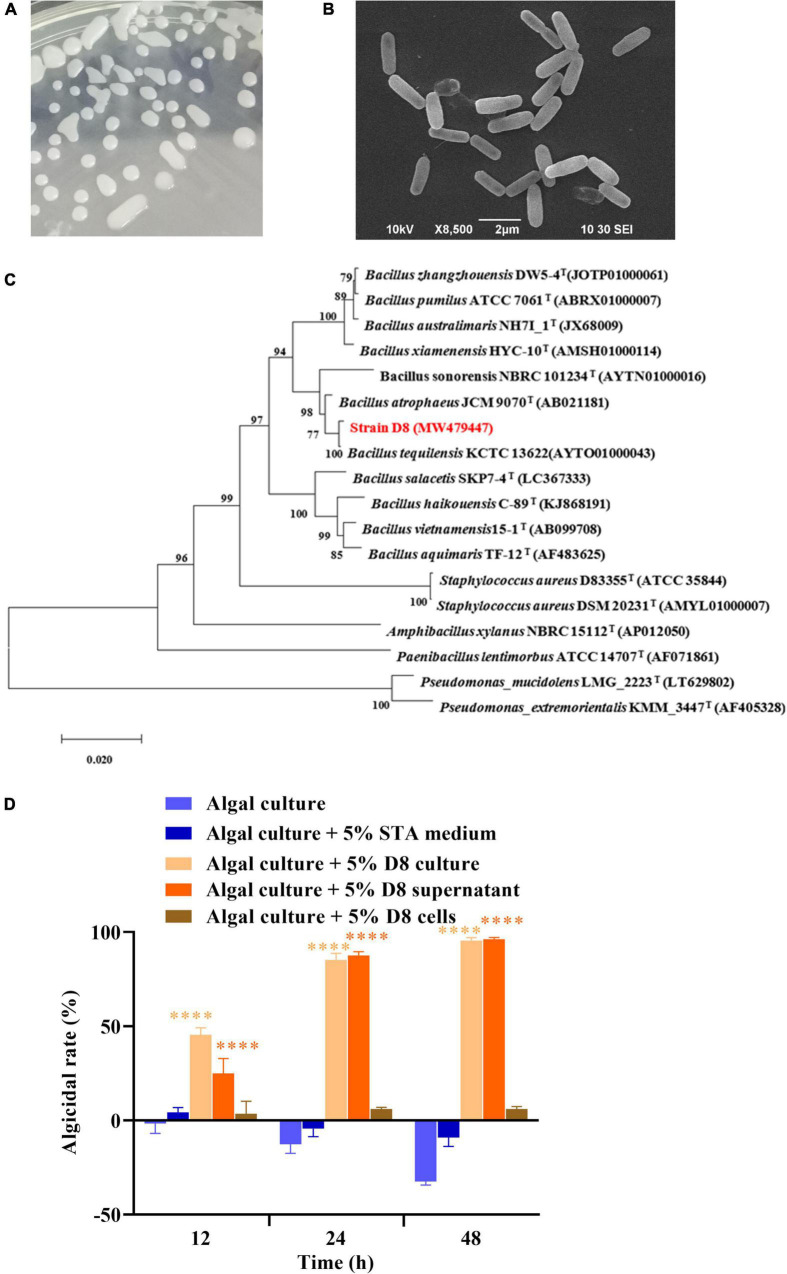
Identification and algicidal activities of *B. tequilensis* D8. **(A)** Strain D8 colonies growing on an STA plate. **(B)** Morphology of strain D8 under SEM. **(C)** Phylogenetic tree of strain D8. **(D)** Algicidal activities and modes of strain D8. Values are the means ± SDs (*n* = 3). ****p < 0.0001 represent significant differences from the control.

Morphological identification showed that the algicidal strain D8 formed white and smooth colonies when plated. SEM observation showed that the cells of strain D8 were short and rod-shaped and lacked flagella (Figures 1A,B). PCR amplification of the 16S rRNA gene (1,546 bp, GenBank accession number: MW479447) and sequencing revealed that D8 shared the highest similarity (99.86%) with *B. tequilensis* KCTC 13622*^T^* (Figure 1C). The results indicated that strain D8 belonged to the genus *Bacillus*, and thus we named it *B. tequilensis* D8. The algicidal activity analysis showed that both the D8 culture and the cell-free supernatant exhibited strong algicidal activities (above 85%) against *H. akashiwo* after treatment for 24 h, and the activities exceeded 95% after treatment for 48 h (Figure 1D). The bacterial cells alone exhibited less than 6% algicidal activity. This result indicates that strain D8 killed algal cells by secreting active substances into the supernatant.

In addition to *H. akashiwo*, strain D8 showed high algicidal activities against *Prorocentrum donghaiense, Thalassiosira pseudonana*, and *Skeletonema costatum*. However, it exhibited low algicidal activities against species belonging to Cyanophyta and did not kill species belonging to Chlorophyta (Supplementary Table 1).

The stability of the algicidal activity of the D8 supernatant was evaluated at various temperatures and light intensities. Supplementary Figure 1 shows that the algicidal activity of the D8 supernatant was stable from −80 to 121°C and was not affected by light exposure. The results suggest that the algicidal substances produced by strain D8 exhibit good temperature and light stability and have potential applications in the biocontrol of HABs.

### Algicidal effects of different concentrations of the D8 crude extract

To explore the chemical nature of the algicidal substances, the D8 supernatant was first precipitated by HCl or NaOH. The precipitate and supernatant were then separately collected by centrifugation. Supplementary Figure 2 shows that only the 5% D8 supernatant and 5% acid precipitate exhibited obvious algicidal activities, while the 5% HCl-precipitated supernatant, 5% alkali precipitate, and 5% NaOH-precipitated supernatant showed no significant algicidal effects, indicating that the main algicidal substances were present in the acid precipitate.

**FIGURE 2:**
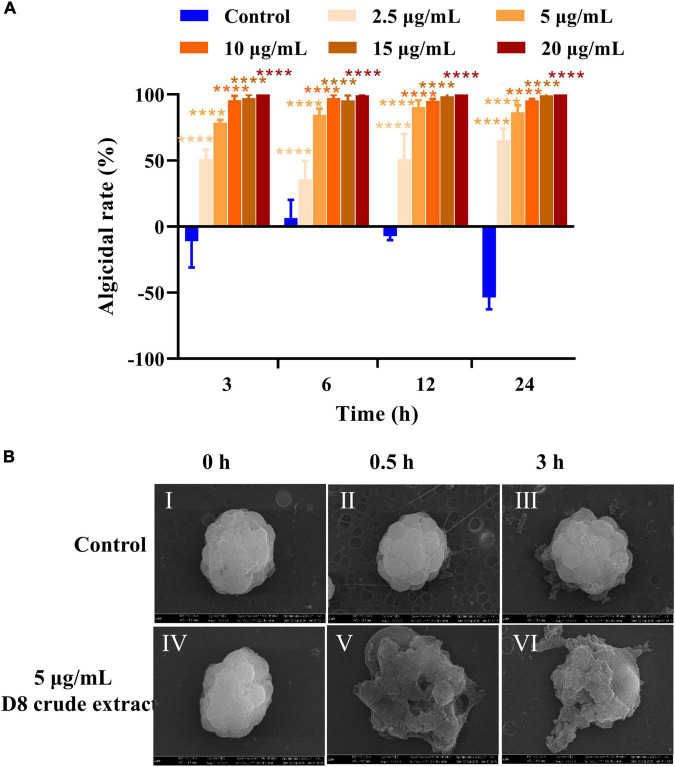
Algicidal activities and effect of D8 crude extract from *B. tequilensis* D8. **(A)** Algicidal activity of different concentrations of D8 crude extract. ****p < 0.0001 represent significant differences from the control. **(B)** Effect of the D8 crude extract on algal cell morphology. Algal cells treated with DMSO for 0 h (I), 0.5 h (II), and 3 h (III) and with the D8 crude extract for 0 h (IV), 0.5 (V), and 3 h (VI).

The acid precipitate was further purified to obtain the D8 crude extract. The algicidal activity measurements showed that treatment with 2.5, 5, 10, 15, and 20 μg/ml D8 crude extract resulted in algicidal rates of 57.4, 78.5, 95.7, 97.2, and 100%, respectively, after 3 h (Figure 2A), indicating that the D8 crude extract had strong algicidal activity against *H. akashiwo* in a short period of time and that the algicidal effect of the D8 crude extract was concentration dependent.

### Morphological effect of the D8 crude extract on *Heterosigma akashiwo*

To monitor the events involved in the cell damage to *H. akashiwo* induced by the D8 crude extract, the morphological changes of the algal cells were observed using SEM. The algal cells in the control group were intact, and the plastids were arranged neatly on the cell surface (Figure 2B). After treatment with the D8 crude extract for 0.5 and 3 h, the algal cell morphology was severely damaged, the cell membrane was ruptured, the plastids had spilled out, and finally, whole cells collapsed (Figure 2B; Supplementary Video 1). When the algal cells were treated with 10 μg/ml D8 crude extract, the resulting dynamic changes showed that the membrane was attacked and rapidly disintegrated, and the cell contents were immediately released (Supplementary Video 1). The above results suggested that the D8 crude extract could rapidly target the cell membrane and lyse algal cells in a short time.

### Effect of the D8 crude extract on the viability of *Heterosigma akashiwo*

**FIGURE 3:**
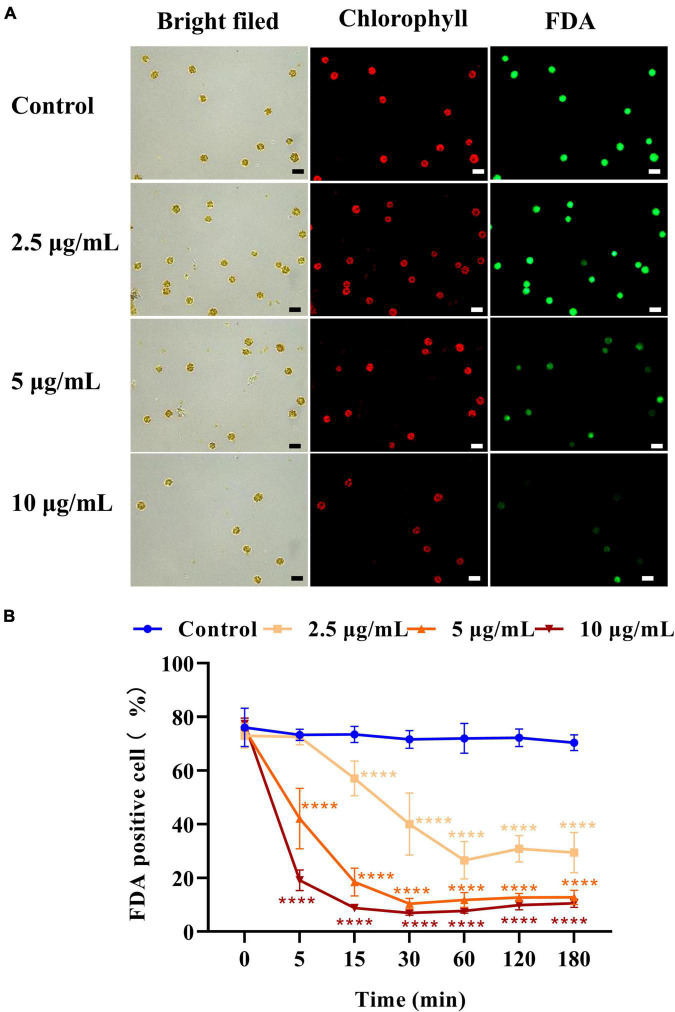
Effect of D8 crude extract on the cell viability of *Heterosigma akashiwo*. Fluorescence microscopy image of algal cells treated with the D8 crude extract for 5 min **(A)** and the proportions of FDA fluorescence positive cells exposed to different concentrations of the D8 crude extract **(B)**. The scale bar represents 20 μm. ****p < 0.0001 represent significant differences from the control.

Fluorescein diacetate (FDA) was used to assess the viability and membrane permeability of *H. akashiwo*. As shown in Figure 3A, the control emitted strong fluorescence, while the fluorescence intensity of algal cells treated with 5 or 10 μg/ml D8 crude extract for 5 min was significantly weaker than that of the control. The results indicated that the D8 crude extract instantly damaged the plasma membrane and eliminated esterase activity. Figure 3B shows that the proportions of fluorescence-positive cells decreased by 71.6, 83.6, and 89.3% after exposure to 2.5, 5, and 10 μg/ml D8 crude extract for 60 min, respectively. This result indicated that the proportion of damaged cells increased significantly with increasing concentrations of D8 crude extract.

### Effect of the D8 crude extract on the photosynthetic system of *Heterosigma akashiwo*

**FIGURE 4:**
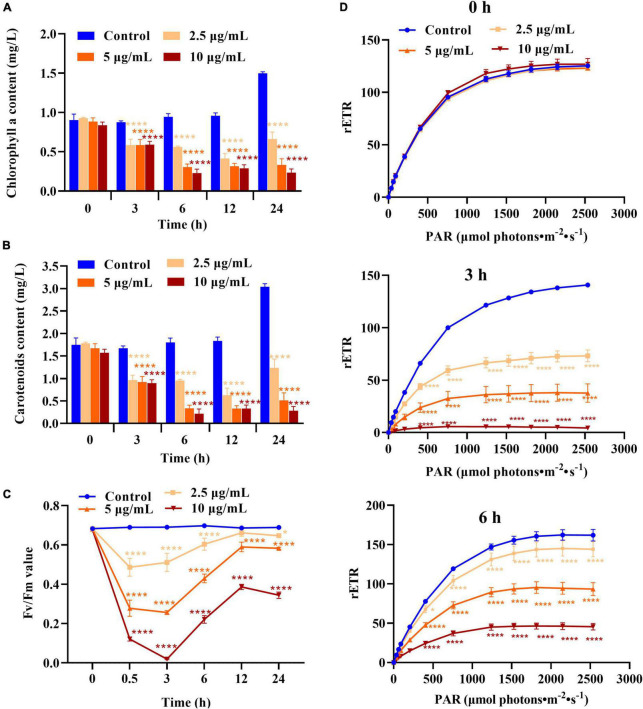
Effect of the D8 crude extract on the photosynthetic system of *Heterosigma akashiwo*. **(A)** Chlorophyll a, **(B)** carotenoid contents, **(C)** Fv/Fm, **(D)** rETR values after treatment with the D8 crude extract. *p < 0.05 and ****p < 0.0001 represent significant differences from the control. ****p < 0.0001 represent significant differences from the control.

According to the above results, the D8 crude extract primarily targets the membrane system of *H. akashiwo*. Photosynthetic electron transformation is dependent on the intact thylakoid membrane. To explore the effect of the D8 crude extract on photosynthesis in *H. akashiwo*, the Chl *a* content, carotenoid content, Fv/Fm and rETR were investigated. As shown in Figures 4A,B, the Chl *a* and carotenoid levels in the algal cells were markedly decreased after treatment with different concentrations of the D8 crude extract for 3, 6, and 12 h and remained at low levels after 24 h of treatment with 5 and 10 μg/ml D8 crude extract; however, the Chl *a* and carotenoid levels began to recover after 24 h of treatment with 2.5 μg/ml D8 crude extract, suggesting that the toxicity of the D8 crude extract was attenuated and the photosynthetic activity of the remaining algal cells resumed.

The Fv/Fm and rETR values of *H. akashiwo* cells treated with D8 crude extract showed similar changes. After treatment for 0.5 and 3 h, the Fv/Fm and rETR values of all the treatment groups decreased markedly, especially after treatment with 10 μg/ml D8 crude extract. The Fv/Fm values decreased to only 3% of that of the control, and the rETR values were less than 5% of that of the control, indicating that photosynthesis in the algal cells was damaged. However, with increasing treatment time, the Fv/Fm and rETR values of algal cells in all the treatment groups recovered significantly, and the recovery rate of the low-concentration group was faster than that of the high-concentration group. The rETR values of algal cells in the 2.5, 5, and 10 μg/ml treatment groups recovered to approximately 90, 60, and 30% of that of the control, respectively, after treatment for 6 h (Figures 4C,D). The Fv/Fm values of the 2.5, 5, and 10 μg/ml treatment groups recovered to 94, 86, and 56% of that of the control, respectively, after treatment for 12 h. The results suggested that the D8 crude extract could dramatically inhibit and destroy the photosynthesis of *H. akashiwo*, but the remaining algal cells could self-restore and recover their photosynthetic function after the toxicity of the D8 crude extract was attenuated.

### Identification of algicidal compounds

An oil drain ring showed that there was high correlation between the concentration of the D8 crude extract and the diameter of the oil drain ring (Supplementary Figure 3), indicating that there were biosurfactants in the D8 crude extract. To further identify the substances in the D8 crude extract that played a role in algal cell lysis, we used HPLC to isolate and purify algicidal components. As shown in Supplementary Figure 4, the D8 crude extract contained 4 main components. Algicidal experiments showed that components 2, 3, and 4 had obvious algicidal effects, but component 1 did not. According to the molecular weights in Supplementary Figure 4, components 2, 3, and 4 may be a class of homologues, as the differences between the molecular weights of the three substances are 14, which is equivalent to the mass of a CH_2_ group.

**FIGURE 5:**
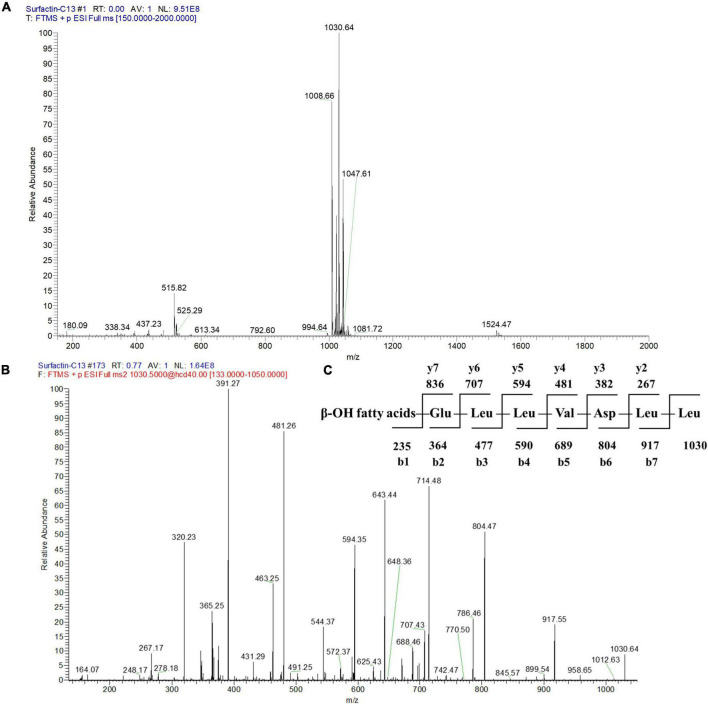
Mass spectrogram of component 2. Primary mass spectrometry **(A)**, secondary mass spectrometry **(B)** of the [M + Na]^+^ ion peak at m/z = 1030.64 and secondary mass spectrometry fragmentation rule **(C)** of component 2.

**FIGURE 6:**
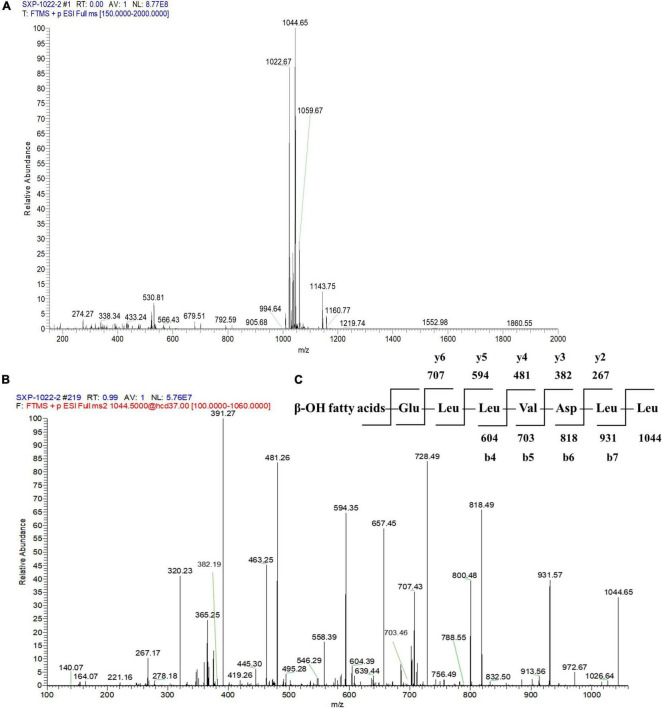
Mass spectrogram of component 3. Primary mass spectrometry **(A)**, secondary mass spectrometry **(B)** of the [M + Na]^+^ ion peak at m/z = 1044.65 and secondary mass spectrometry fragmentation rule **(C)** of component 3.

**FIGURE 7:**
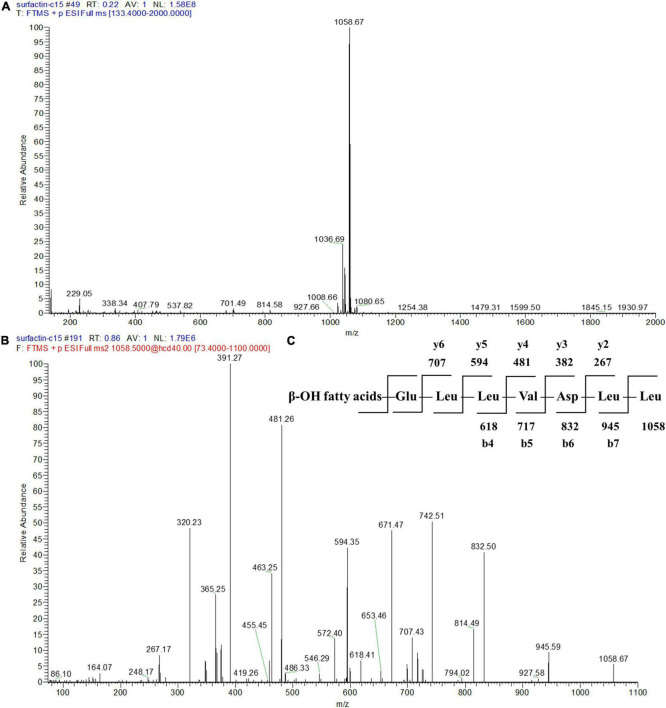
Mass spectrogram of component 4. Primary mass spectrometry **(A)**, secondary mass spectrometry **(B)** of the [M + Na]^+^ ion peak at m/z = 1058.67 and secondary mass spectrometry fragmentation rule **(C)** of component 4.

To further identify the structures of the three components, we used Q-Exactive high-resolution liquid chromatography-mass spectrometry to explore their specific structures. The full scan mass spectrometry (MS1) of the three compounds showed a series of ion peaks of [M + H]^+^ (at m/z 1008, 1022, and 1036) and [M + Na]^+^ (at m/z 1030, 1044, and 1058) (Figures 5A, 6A, 7A). Interestingly, these ion data were very similar to those reported for surfactin-type lipopeptides in *Bacillus*. However, due to the existence of many surfactin isoforms, secondary mass spectrometry (MS2) was used to further understand the amino acid compositions of these three components. The [M + Na]^+^ ion peak was chosen for further tandem mass spectrometry analysis. The MS2 spectra of the three compounds showed a series of ion peaks that indicate characteristic surfactin-type peptide fragmentations (Figures 5B, 6B, 7B). The MS2 data of these compounds generated a series of y-type ions and b-type ions, allowing amino acid sequence analysis. The series of y^+^ ions at m/z 836→707→594→481→382→267 represented cleavage along the peptide bonds due to the loss of Glu, Leu, Leu, Val, and Asp from the C-terminus of a C13 β-OH fatty acid, whereas the b^+^ ions at m/z 1030→917→804→689→590→477→364→235 indicated the loss of Leu, Leu, Asp, Val, Leu, Leu, Leu, and Glu from the N-terminus (Figures 5C, 6C, 7C; Supplementary Table 2). According to the rules of cleavage, the amino acid compositions of the three components were identical. Therefore, it could be inferred that the amino acid sequence, starting from the C-terminus, was Glu_1_, Leu/IIe_2_, Leu/IIe_3_, Val_4_, Asp_5_, Leu/IIe_6_, and Leu/IIe_7_. However, because leucine and isoleucine have the same molecular weight, NMR technology was needed to further determine the amino acid composition.

### Nuclear magnetic resonance-based identification of three algicidal compounds

Although the molecular weights of leucine and isoleucine are the same, their terminal methyl positions are different, so they exhibit obvious differences in their one-dimensional NMR spectra. In the ^13^C NMR spectrum, the chemical shift of the terminal methyl group in leucine was approximately 21–23 ppm, while that of isoleucine was significantly different: the chemical shift of one methyl group was 11.2 ppm, while that of the other was 15.6 ppm. The ^13^C NMR spectra (Supplementary Table 3 and Supplementary Figures 5–7) of the three algicidal components showed that they all had only peaks with chemical shifts of 22-23 ppm but no peaks with chemical shifts of 11.2 and 15.6 ppm, which suggested that they contained 4 leucines but no isoleucine. In addition, the δ_*C*_ of 169.4–174.4 ppm indicated representative signals for carboxyl groups.

**FIGURE 8:**
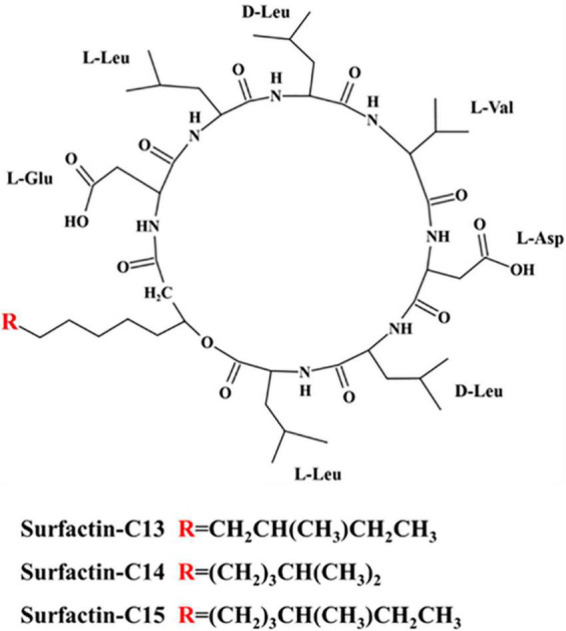
Chemical structures of the three algicidal compounds.

We also found some characteristic peaks in the ^1^H-NMR spectra (Supplementary Table 3 and Supplementary Figures 5–7). More specifically, these data included a δ_*H*_ of 1.20 ppm for a broad singlet peak for a fatty acid chain, δ_*H*_ of 4.04–4.95 ppm for multiplet peaks for the C_α_ of amino acids, and δ_*H*_ of 7.76–8.43 ppm for singlet and doublet peaks for “-NH-” units. By combining the data of the ^1^H-NMR spectra, ^13^C-NMR spectra and mass spectra and referring to related articles (Tang et al., 2007; Ma and Hu, 2015), the chemical shifts of the ^1^H-NMR spectra and ^13^C-NMR spectra of the three algicidal components were collected and are shown in Table 1. In summary, we elucidated that the three components were surfactin-C13, surfactin-C14 and surfactin-C15. The chemical structures of these three algicidal components are shown in Figure 8.

**TABLE 1:** Half-lethal concentration (LC_50_, μg/mL) of surfactin homologues for *Heterosigma akashiwo, Skeletonema costatum*, and *Prorocentrum donghaiense*.

Algal species	*Heterosigma akashiwo*		*Skeletonema costatum*		*Prorocentrum donghaiense*	
Time (h)	12	24	12	24	12	24
Surfactin-C13	1.68	1.22	2.62	2.35	2.34	2.3
Surfactin-C14	1.53	1.2	2.66	1.52	14.82	5.3
Surfactin-C15	57.24	89.11	3.6	1.71	**—**	**—**

### Algicidal activities of surfactin-C13, surfactin-C14, and surfactin-C15

To evaluate the algicidal activities of the three surfactin homologues, we measured their LC_50_ values against three HAB-causing species, namely, *H. akashiwo, S. costatum*, and *P. donghaiense*. The results (Table 1) showed that surfactin-C13 and surfactin-C14 exhibited high algicidal activities against the three algal species. The LC_50_ values of surfactin-C13 for the three algal species were less than 3 μg/ml. The LC_50_ values of surfactin-C14 for *H. akashiwo* and *S. costatum* were less than 3 μg/ml, while the value for *P. donghaiense* was higher than 5 μg/ml. The 12 h-LC_50_ of surfactin-C14 for *P. donghaiense* was 14.82 μg/ml, and the 24 h-LC_50_ was 5.31 μg/ml. The algicidal activities of surfactin-C15 were weaker than those of surfactin-C13 and surfactin-C14. Surfactin-C15 had high algicidal activity against *S. costatum* and no algicidal effect on *P. donghaiense*. Although surfactin-C15 had an algicidal effect on *H. akashiwo*, the LC_50_ value was higher than 50 μg/ml. The results suggested that the fatty acid chain length probably changed the algicidal activity of the surfactin homologues. *S. costatum* was the most sensitive to all three surfactins.

## Discussion

Harmful algal blooms have caused global disasters that endanger marine ecosystems and human health (Grattan et al., 2016). It is urgent to find a safe and effective treatment method for controlling HABs. The high efficiency and specificity of algicidal bacteria have attracted extensive attention from researchers (Pal et al., 2020). Known algicidal bacteria include *Cytophaga* (Mayali and Azam, 2004), *Pseudomonas* (Kim et al., 2018), *Vibrio* (Wang et al., 2020c), *Flavobacterium* (Wang et al., 2020a), and *Hahella* (Zhang et al., 2020) species. In this study, *B. tequilensis* strain D8 was found to exert a strong algicidal effect on *H. akashiwo*, a dominant HAB-causing species, by producing algicidal extracellular substances. In addition to its effect on *H. akashiwo*, the algicidal substances showed high algicidal activity against *T. pseudonana* and *S. costatum* in Bacillariophyta and *P. donghaiense* in Pyrrophyta and low algicidal activity against *M. aeruginosa* and *Synechocystis* sp. in Cyanophyta. However, the algicidal substances did not lyse *P. subcordiformis, P. helgolandica, C. vulgaris*, or *D. salina* in Chlorophyta. We speculated that green algae and cyanobacteria are less sensitive to algicidal compounds due to their cell wall structure. Moreover, the algicidal substance exhibited stable algal-lytic activities under different temperatures (−80 to 120°C) and light intensities (0–1,000 μmol photons m^–2^ s^–1^). The stability of algicidal compounds under various temperature and light exposure conditions indicates their practical advantage under temporarily adverse environmental conditions, which could facilitate their potential application in eliminating HABs. The algal-lytic process observation indicated that the algicidal compounds primarily targeted cell plasma membranes. The rapid significant decreases in the Fv/Fm and rETR values also suggested damage to the thylakoid membranes of *H. akashiwo* after treatment with D8 algicidal compounds. Wang et al. (2005) reported that a rhamnolipid produced by *P. aeruginosa* caused rapid lysis of *H. akashiwo*, and the reported algal-lytic process and characteristics of the rhamnolipid were similar to the algicidal effect of the D8 crude extract from *B. tequilensis*. Due to its amphiphilicity, surface activity and capacity to rapidly and strongly attack cell membranes, the algicidal substances of *B. tequilensis* D8 were speculated to be surfactants. Furthermore, surfactants were found to inhibit the lipolytic efficiency of lipase (a kind of esterase) by generating inactive aqueous enzyme-surfactant complexes by blocking the accumulation of enzymes at the lipid/water interface (Delorme et al., 2011; Chen et al., 2020). FDA fluorescent staining indicated that the D8 algicidal compounds decreased the activity of intracellular esterases, indicating that the algicidal compounds of strain D8 may be surfactants.

Cao and Yu (2003) elucidated that the excellent algicidal effect of hexadecyl trimethyl ammonium bromide, a chemical surfactant, on *H. akashiwo* was mainly due to its powerful surfactivity and tendency to accumulate on the phospholipid biomolecular surface of cells, followed by weakening of the organelles. Biosurfactants, such as rhamnolipids, sophorolipids, and iturin homologues, have been reported to exhibit high algicidal activity against *H. akashiwo*. Both 4 mg/l rhamnolipids (Wang et al., 2005) and 10 mg/l sophorolipids (Sun et al., 2004) killed more than 90% of *H. akashiwo* cells. *Bacillus* species can synthesize a mixture of amphiphilic lipopeptides by non-ribosomal peptide synthetases, which mainly include members of the surfactin, iturin, lichemysin, and fengycin families with broad-spectrum biological activities. Each family of lipopeptides produced by a particular strain varies in accordance with the culture conditions (Lv et al., 2020). Sang et al. (2006) found that three iturin homologues (m/z = 1056, 1070, and 1084) showed algicidal activities against the dinoflagellate *Cochlodinium polykrikoidesis* with LC_50_ values (6 h) of 2.3, 0.8, and 0.6 μg/ml, respectively. However, the surfactin and fengycin families have seldom been reported to have algicidal activity. Although Ahn et al. (2003) reported that the culture broth of *B. subtilis* C1 containing surfactin completely inhibited the growth of the cyanobacterium *M. aeruginosa*, and they speculated that the damage to *M. aeruginosa* was caused mainly by destabilization of the membranes by the surfactin, they did not isolate the biosurfactant surfactin from the culture broth to demonstrate its algicidal activity. Furthermore, the surfactin exhibited only very weak algicidal activities against species of Cyanophyta species, including *M. aeruginosa*, according to its algicidal spectrum. Therefore, we speculated that other active compounds in the culture broth of *B. subtilis* C1, not surfactins, harbor algicidal activity against *M. aeruginosa.* In this study, we isolated three algicidal substances from *B. tequilensis* D8 and identified them as lipopeptide surfactin homologues, namely, surfactin-C13, surfactin-C14, and surfactin-C15, by interpreting MS and NMR spectroscopy data. This is the first report of *B. tequilensis* lysing *H. akashiwo, S. costatum*, and *P. donghaiense* by producing surfactins. Based on differences in their structures and bioactivities, the chain length of the fatty acid moiety of surfactin might have affected the algicidal activity by influencing the lipophilicity of the compound. Surfactin-C13 has a shorter fatty acid chain than surfactin-C14 and surfactin-C15 and exerted higher algicidal activity against three HAB-causing species, *H. akashiwo, S. costatum*, and *P. donghaiense*, with LC_50_ values of 1.22–2.62 μg/ml. Surfactin-C14 showed LC_50_ values of 1.2–14.82 μg/ml. Surfactin-C15 showed weak algicidal activity against *S. costatum* and *H. akashiwo* with LC_50_ values of 1.71–89.11 μg/ml but exhibited little activity against *P. donghaiense*. Thus, surfactin-C13 and C-14 exhibited higher algicidal activities than surfactin-C15 and were more suitable for the control of HABs caused by *H. akashiwo, S. costatum*, and *P. donghaiense*.

It is well-known that surfactants, even at very low concentrations, can bind to cell membranes, affecting membrane permeability. At higher concentrations, more drastic effects, such as membrane lysis and fusion, have been reported (De Oliveira et al., 2017). In the present study, surfactin promoted *H. akashiwo* swelling and caused cell lysis, which indicated modification of membrane permeability. We therefore concluded that these three surfactin homologues from the D8 strain, which are amphiphilic cyclic lipopeptides and are actually regarded as a family of biosurfactants, could easily accumulate on the phospholipid bilayer surfaces of algal cells and alter the phospholipid bilayers, facilitating the entry of surfactins and other substances. Then, the esterase activities and membrane systems of other organelles, such as the thylakoid membrane, were destroyed by phagocytosed surfactins. Chen et al. (2020) reported that the lipopeptides fengycin, iturin, and surfactin exhibited strong and dose-dependent inhibitory activities against lipase. We also found that, after entry, the surfactins rapidly inhibited esterase activity, as shown by FDA staining. Furthermore, the three surfactins showed selective toxicity to three HAB-causing species. *S. costatum* was the most sensitive to all three surfactins and *P. donghaiense* showed weak sensitivity to surfactin-C14 and little sensitivity to surfactin-C15. The algicidal mechanism depends on the characteristics of the compound, and the effects are different depending on the target organism. Several studies have shown that the sensitivity of lipid membranes to surfactin is mainly determined by the lipid composition, polar head group region, length of fatty acid acyl chain and lipid organization (Kell et al., 2007; Buchoux et al., 2008; Deleu et al., 2013; Wu et al., 2017). *H. akashiwo, S. costatum* and *P. donghaiense* showed different sensitivities to surfactins probably related to the lipid composition, length of the fatty acid acyl chain and lipid organization of their lipid membrane.

Knowledge about the toxicity of surfactins is essential for the use of these compounds to control HABs. Surfactins have been reported to be susceptible to chemical reactions and degradation under physiological conditions due to the presence of aspartic acid-glycine segments in their peptide moieties (Seydlová and Svobodová, 2008; De Oliveira et al., 2017). De Oliveira et al. (2017) reported that crude surfactin extract (less than 100 μg/ml) was degraded by more than 65% within 72 h by both *Pseudomonas putida* and a mixed microbial community from a sewage-treatment plant, and the biodegradation percentages exceeded the biodegradation established by the Organization for Economic Co-operation and Development (OECD, Guidelines 301E). Therefore, surfactins can be classified as readily biodegradable compounds. Furthermore, acute toxicity measurements carried out with *Vibrio fischeri, Daphnia magna*, and *Selenastrum capricornutum* indicated that the toxicity of surfactins was lower than that reported for conventional surfactants. The crude surfactin concentration needed to produce 50% growth inhibition (EC_50_-72 h) of *S. capricornutum* was 49.3 μg/ml, and the EC_50_-48 h value of surfactin against *D. magna* growth was as high as 170.1 μg/ml. The EC_50_-30 min of surfactin against *V. fischeri* light emission was 848.2 μg/ml (De Oliveira et al., 2017). These reported EC_50_ values are much higher than the LC_50_ values of surfactins needed to produce 50% cell death in three HAB-causing species in the present study. Recently, we tested the onsite algicidal activity and acute toxicity of surfactin-C13 and surfactin-C14 (5–10 μg/ml) against *Photobacterium phosphreum* T3 spp., *Artemia salina*, and juvenile *Lateolabrax japonicus* fish. The experimental data indicated that 5 μg/ml surfactin-C13 or surfactin-C14 lysed more than 90% of algal cells and showed less toxicity to *Photobacterium phosphreum* T3 spp., *Artemia salina*, and *Lateolabrax japonicus*. Therefore, surfactin has great potential in controlling, reducing, and eliminating HABs due to its high algicidal activity, low toxicity, and degradability.

## Conclusion

*Bacillus tequilensis* D8, which was isolated from the seawater of a coastal HAB area, lyses *H. akashiwo* by producing three surfactin homologues and probably plays an important role in decreasing and eliminating *H. akashiwo* blooms. The algicidal activity of surfactin-C13 and surfactin-C14 was higher than that of surfactin-C15. The surfactins damaged the plasma membrane and destroyed thylakoid membrane function, thereby rapidly lysing algal cells. Furthermore, surfactins inhibited esterase activity after entering the treated algal cells. In addition to *H. akashiwo*, the surfactins exhibited strong algicidal activities against *S. costatum* and *P. donghaiense* and can potentially be applied in the control of HABs caused by these three algal species. The algicidal activity of the surfactins was stable under various temperatures (−80 to 121°C) and light intensities. The stability of the surfactins under extreme temperature conditions allows a wide range of potential applications in fields ranging from biotechnology to environmental clean-up.

## Data availability statement

The datasets presented in this study can be found in online repositories. The names of the repository/repositories and accession number(s) can be found below: https://www.ncbi.nlm.nih.gov/genbank/, MW479447.

## Author contributions

XS: methodology, investigation, formal analysis, data curation, visualization, and original writing. WX and YL: investigation and visualization. GL and LL: investigation. WZ and QX: methodology. HX: conceptualization, methodology, writing, supervision, and funding acquisition. All authors contributed to the article and approved the submitted version.

## Footnotes

1
www.ezbiocloud.net
