# Supplementary material for: Algicidal characteristics of novel algicidal compounds, cyclic lipopeptide surfactins from Bacillus tequilensis strain D8, in eliminating Heterosigma akashiwo blooms
Source: Front Microbiol. 2022 Nov 30;13:1066747. doi: 10.3389/fmicb.2022.1066747 (PMC9748430; doi:10.3389/fmicb.2022.1066747)
Supplement: Supplementary file 1 [file Data_Sheet_1.pdf]

Supplementary material

Materials and methods

Algicidal spectrum assay

To investigate the algicidal specificity of strain D8, 13 algal species in addition to *H. akashiwo* were tested by adding 5% (vol/vol) fresh D8 culture, followed by culturing under the conditions described above at 20 ±1°C for 72 h. These additional algal species were *Platymonas subcordiformis*, *Dunaliella salina*, *Platymonas helgolandica*, *Chlorella vulgaris*, *Phaeocystis globosa*, *Prorocentrum donghaiense*, *Alexandrium tamarense*, *Thalassiosira pseudonana*, *Thalassiosira weissflogii*, *Phaeodactylum tricornutum*, *Skeletonema costatum*, *Microcystis aeruginosa* and *Synechocystis* sp. PCC6803, which were provided by the State Key Laboratory of Marine Environmental Science at Xiamen University. The algal cultures were maintained in sterile f/2 medium prepared with natural sea water or BG-11 medium at 20 ±1°C with illumination at a light intensity of 50 μmol photons m^{-2} s^{-1} under a 12:12 h light-dark cycle.

Algicidal stability assay

The cell-free supernatant of the D8 culture was incubated at -80, 4, 30, 50, 60, 70, 80, 90, 100 or 121°C for 2 h, and after returning to room temperature (25°C), the treated supernatants were added to algal cultures at 5% (v/v) to examine the effect of temperature on algicidal activity. To investigate the effect of light on algicidal activity, the D8 supernatant was exposed to light of different intensities (0, 50, 100, 500, 1,000 μmol photons m^{-2} s^{-1}) for 2 h and then added to algal cultures. STA medium with the same treatments as those used for the D8 supernatants served as the control group. The

initial D8 supernatant (25°C, pH 8) without any treatment served as the reference group.

Oil spreading assay

Surfactants have the property of reducing oil film surface tension and forming an oil drain ring in the center of the oil film. To confirm the surface activity of the D8 crude extract, 30 mL of ddH_2O was added to a glass plate with a diameter of 9 cm, 5 mL of paraffin oil mixed with a small amount of Sudan red was slowly added to the water surface, and the oil was gently shaken horizontally to spread evenly on the water surface and form a red oil film. Various concentrations (0.01, 0.1, 0.5, 1.0 mg/mL) of D8 crude extract were gently dripped on the red oil film and then the changes in oil drain rings were observed. DMSO was added as the negative control.

Supplementary Tables and Figures

Supplementary Table 1. Algicidal activity of the D8 supernatant against different algal species.

Algal species	Algicidal activity (%)
**Chlorophyta**	
Chlorella vulgaris	—
Dunaliella salina	—
Platymonas helgolandica	—
Platymonas subcordiformis	—
**Pyrrophyta**	
Alexandrium tamarense	36.81
Prorocentrum donghaiense	88.13
**Bacillariophyta**	
Phaeodactylum tricornutum	
Thalassiosira pseudonana	98.23
Thalassiosira weissflogii	10.14
Skeletonema costatum	92.66
**Chrysophyta**	
Phaeocystis globosa	42.57
**Xanthophyceae**	
Heterosigma akashiwo	96.76
**Cyanophyta**	
Microcystis aeruginosa 7820	19.05
Microcystis aeruginosa 1752	27.58
Synechocystis sp. PCC 6803	1.95

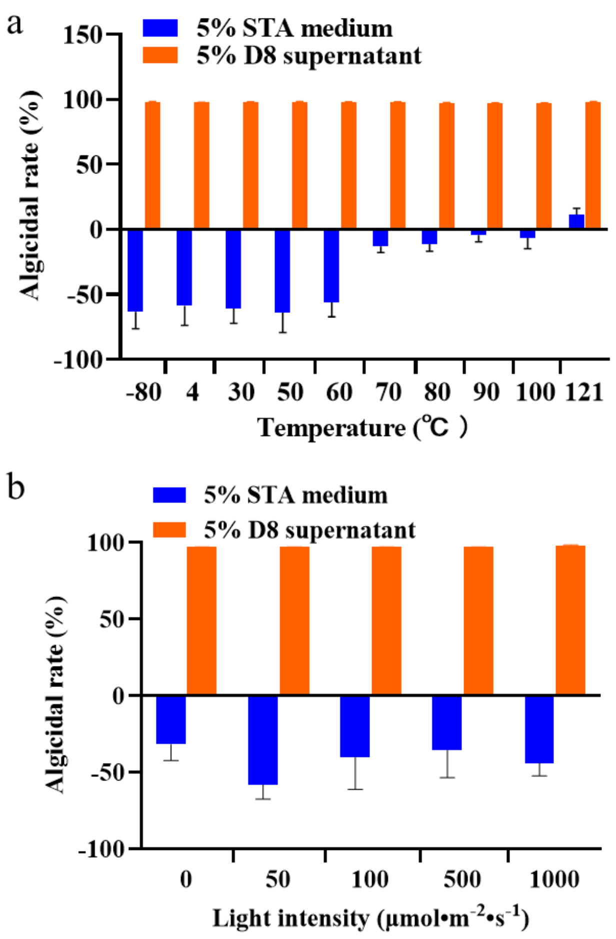

Supplementary Figure 1. Algicidal stabilitiy of the strain D8 supernatant at different temperatures (a) and light intensities (b).

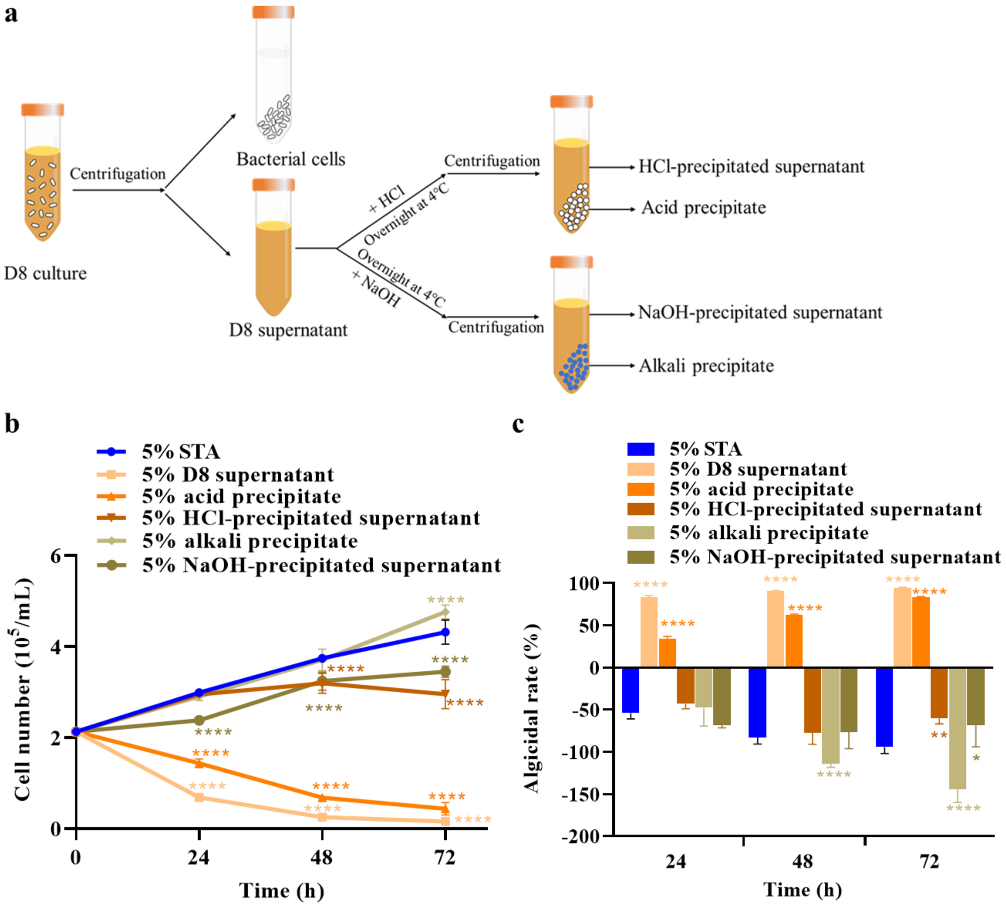

Supplementary Figure 2. Algicidal activities of different components of the D8 supernatant precipitated with acid or alkali. (a) Sketch of the precipitation process with acid or alkali. (b) Cell densities of algal cultures treated with different components of the D8 supernatant. (c) Algicidal rates of different components of the D8 supernatant. * ($p<0.05$), ** ($p<0.01$) and **** ($p<0.0001$) represent significant differences from the control.

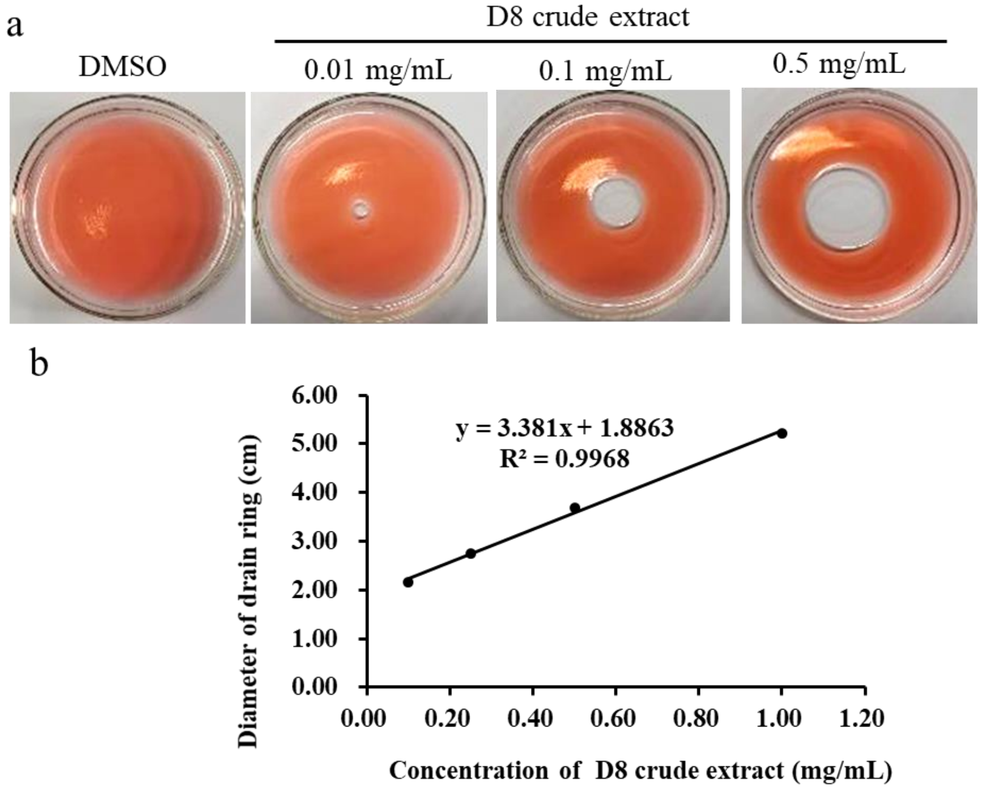

Supplementary Figure 3. Biosurfactant detection of D8 crude extract. (a) Oil drain ring diameters after treatment with different concentrations of D8 crude extract. (b) Linear relationship between D8 crude extract concentrations and oil drain ring diameters.

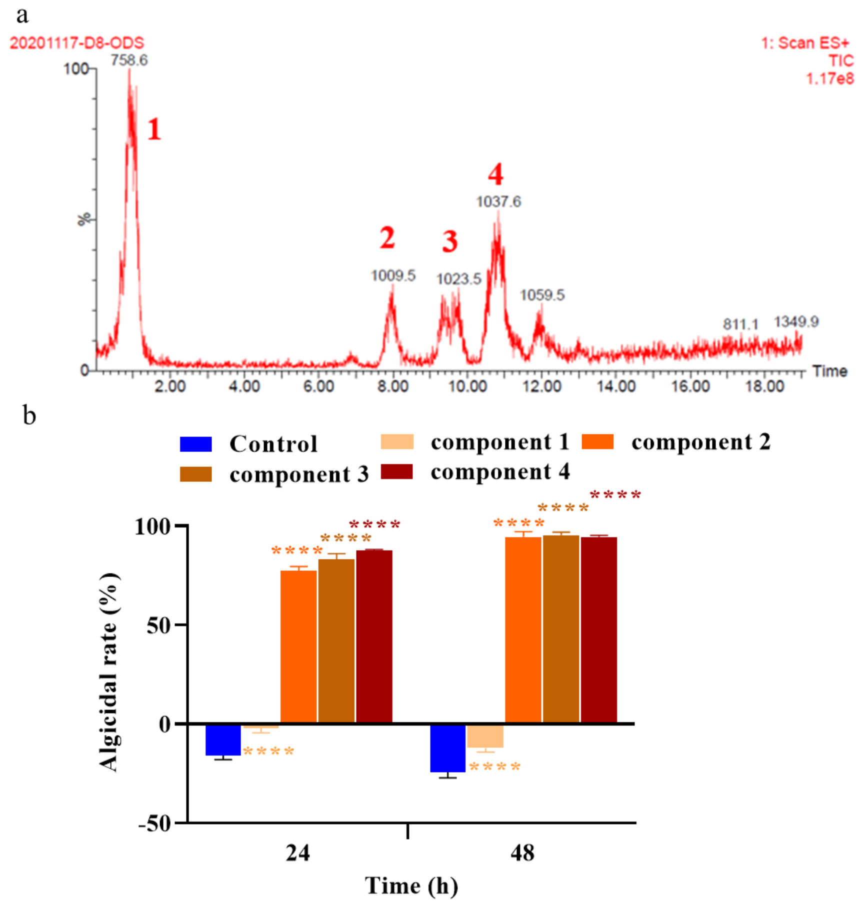

Supplementary Figure 4. HPLC determination (a) and algicidal effect (b) of each component of the D8 crude extract. ** (p<0.0001) represent significant differences from the control.**

a

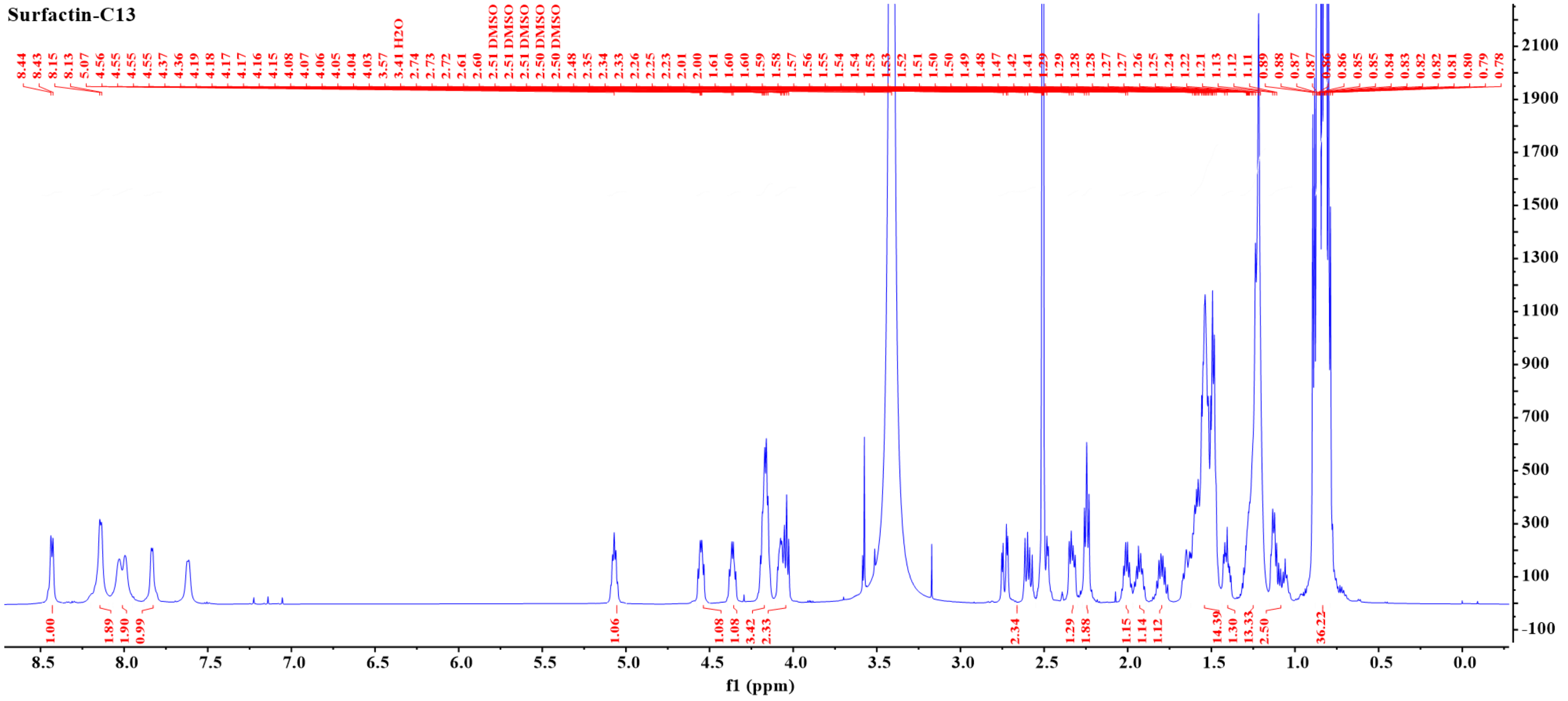
Surfactin-C13
8.44 8.43 8.15 8.13 5.07 4.56 4.55 4.55 4.55 4.37 4.36 4.19 4.18 4.17 4.17 4.16 4.15 4.08 4.07 4.06 4.05 4.04 4.03 3.57 3.41 H2O 2.74 2.73 2.72 2.61 2.60 2.51 DMSO 2.51 DMSO 2.51 DMSO 2.50 DMSO 2.50 DMSO 2.48 2.35 2.34 2.33 2.26 2.25 2.23 2.01 2.00 1.61 1.60 1.60 1.59 1.58 1.57 1.56 1.55 1.54 1.54 1.53 1.53 1.52 1.51 1.50 1.50 1.49 1.48 1.47 1.42 1.41 1.29 1.29 1.28 1.28 1.27 1.27 1.26 1.25 1.24 1.22 1.21 1.13 1.12 1.11 0.89 0.88 0.87 0.87 0.86 0.86 0.85 0.85 0.84 0.83 0.82 0.82 0.81 0.80 0.79 0.78
1.00 1.89 1.90 0.99 1.06 1.08 1.08 3.42 2.33 2.34 1.29 1.88 1.15 1.14 1.12 14.39 1.30 13.33 2.50 36.22
2100 1900 1700 1500 1300 1100 900 700 500 300 100 -100
8.5 8.0 7.5 7.0 6.5 6.0 5.5 5.0 4.5 4.0 3.5 3.0 2.5 2.0 1.5 1.0 0.5 0.0
f1 (ppm)

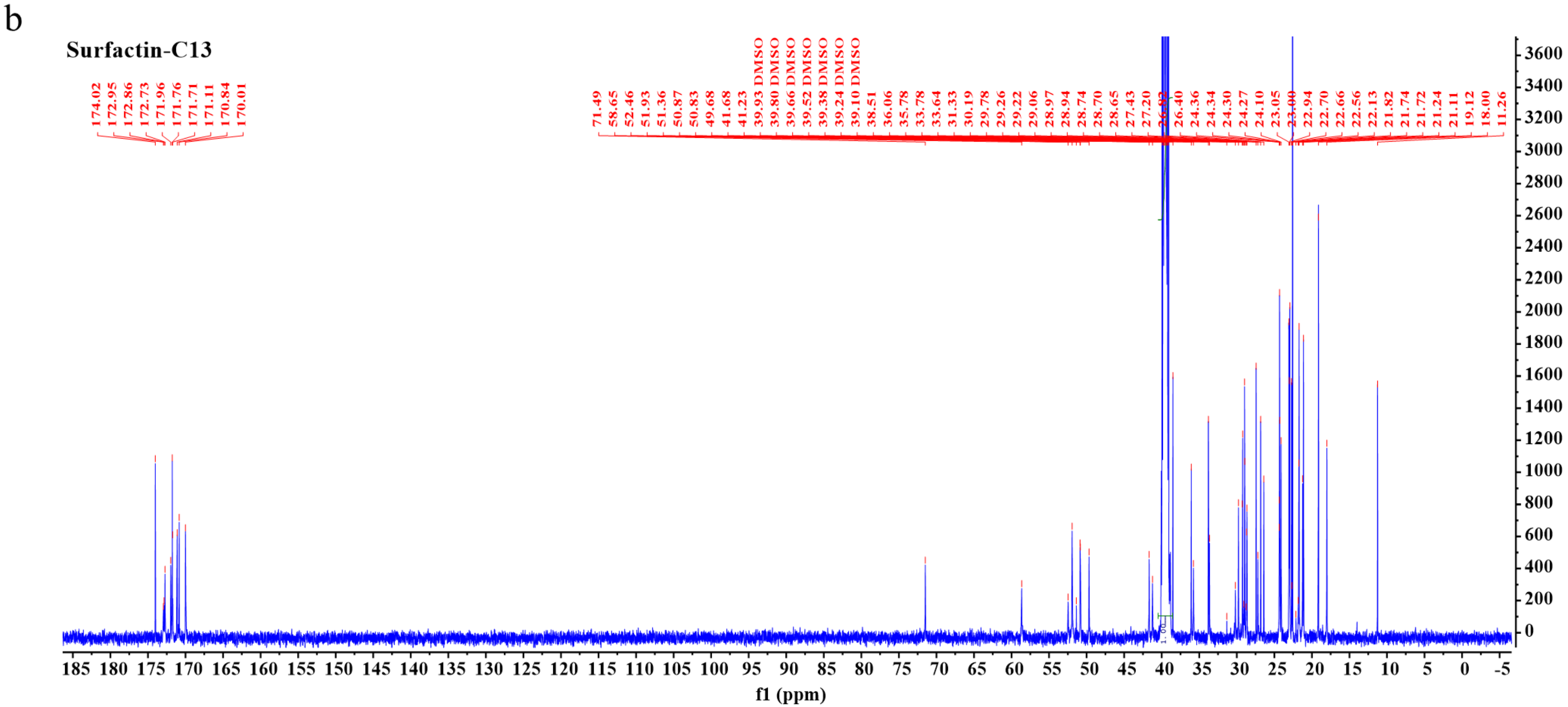

Supplementary Figure 5. ^{1}H-NMR (a) and ^{13}C-NMR (b) spectra of component 2 (surfactin-C13).

a

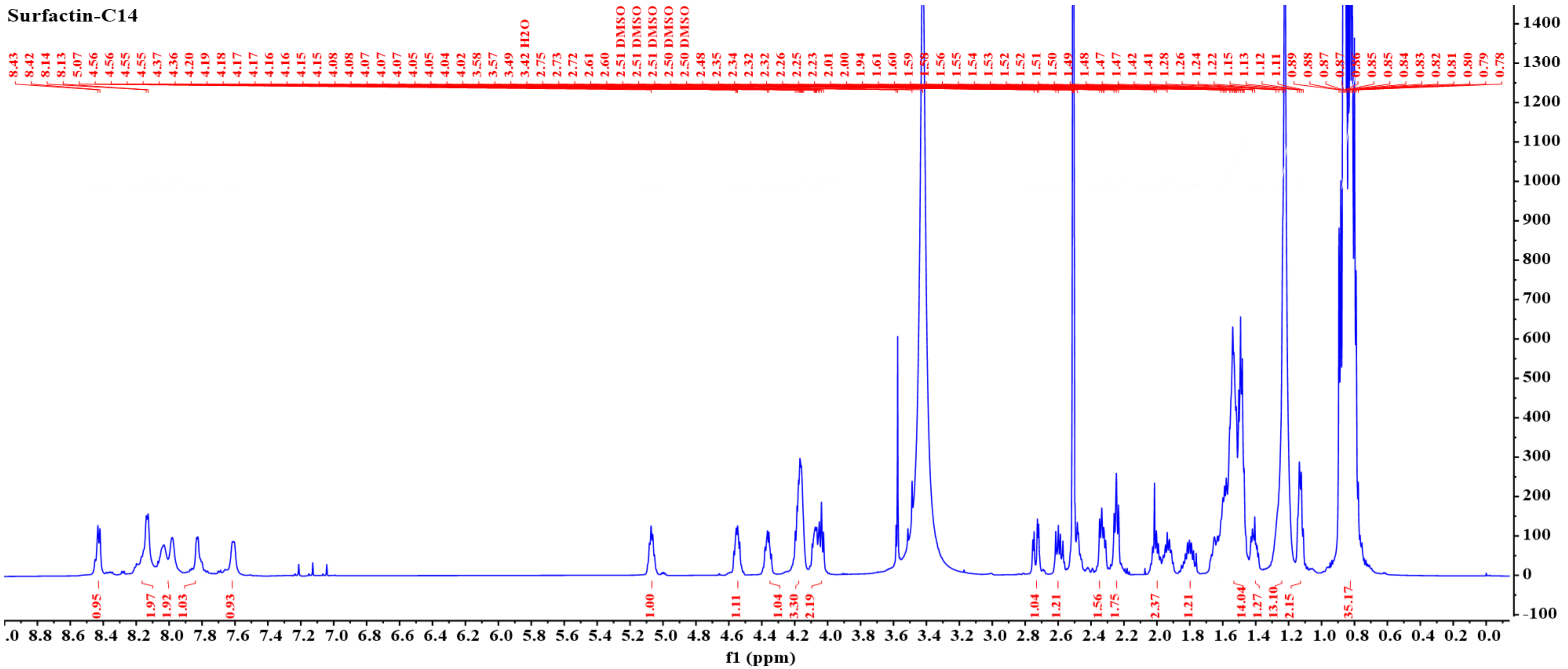

b

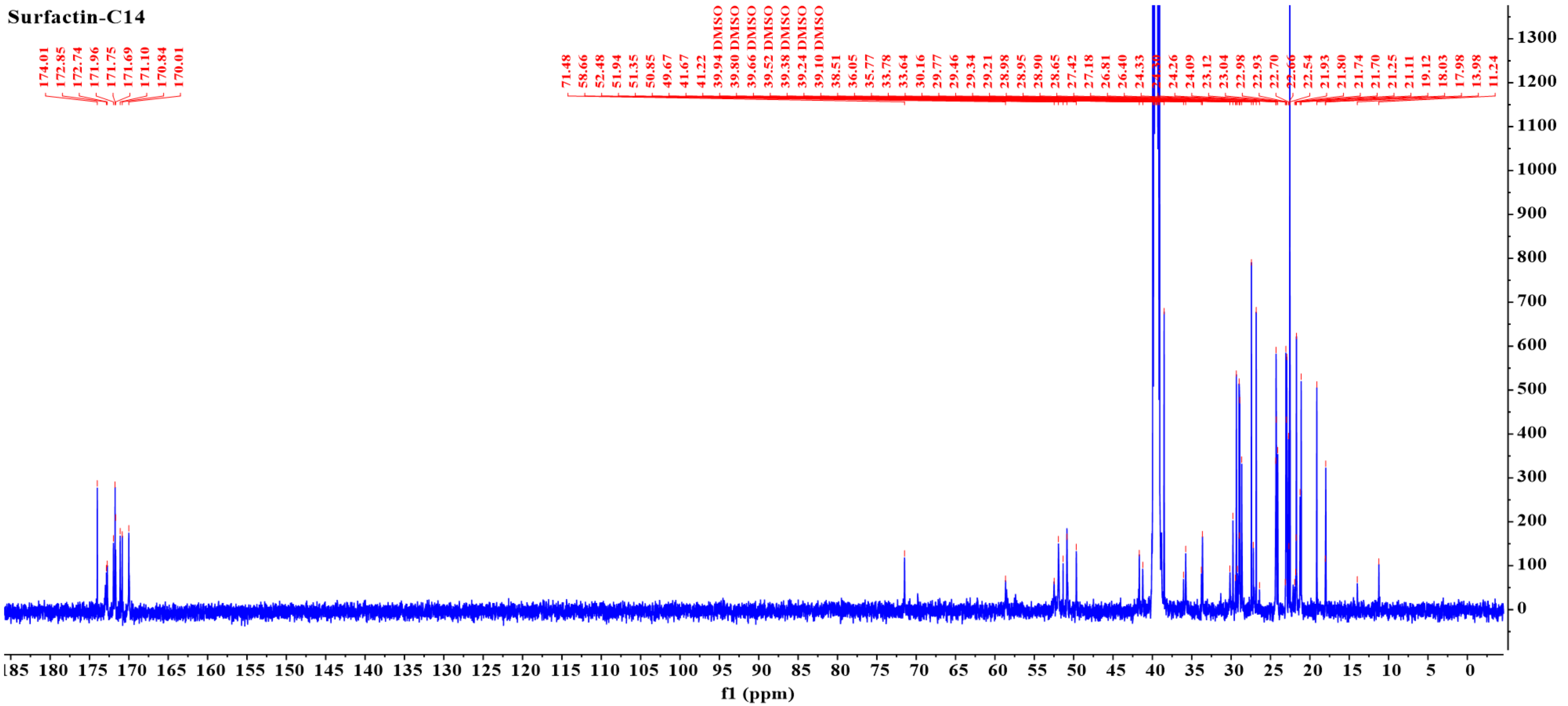

Supplementary Figure 6. ^{1}H-NMR (a) and ^{13}C-NMR (b) spectra of component 3 (surfactin-C14).

a

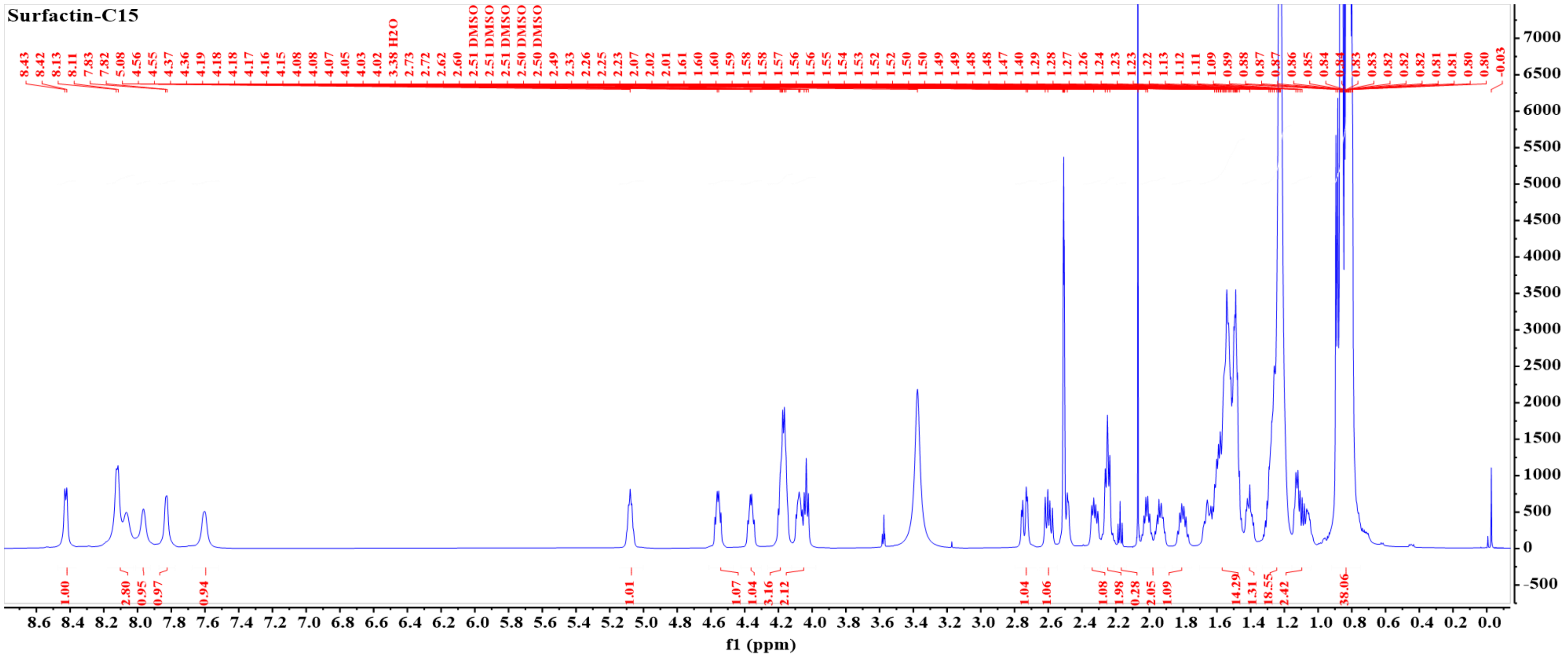
Surfactin-C15
8.43 8.42 8.13 8.11 7.83 7.82 5.08 4.56 4.55 4.37 4.36 4.19 4.18 4.18 4.17 4.16 4.15 4.08 4.08 4.07 4.05 4.03 4.02 3.38 H2O 2.73 2.72 2.62 2.60 2.51 DMSO 2.51 DMSO 2.51 DMSO 2.50 DMSO 2.50 DMSO 2.49 2.33 2.26 2.25 2.23 2.07 2.02 2.01 1.61 1.60 1.60 1.59 1.58 1.58 1.57 1.56 1.56 1.55 1.54 1.53 1.52 1.52 1.50 1.50 1.49 1.49 1.48 1.48 1.47 1.40 1.29 1.28 1.27 1.26 1.24 1.23 1.23 1.22 1.13 1.12 1.11 1.09 0.89 0.88 0.87 0.87 0.86 0.85 0.84 0.84 0.83 0.83 0.82 0.82 0.82 0.81 0.81 0.80 0.80 -0.03
1.00 2.80 0.95 0.97 0.94 1.01 1.07 1.04 3.16 2.12 1.04 1.06 1.08 1.98 0.28 2.05 1.09 14.29 1.31 18.55 2.42 38.06
f1 (ppm)

b

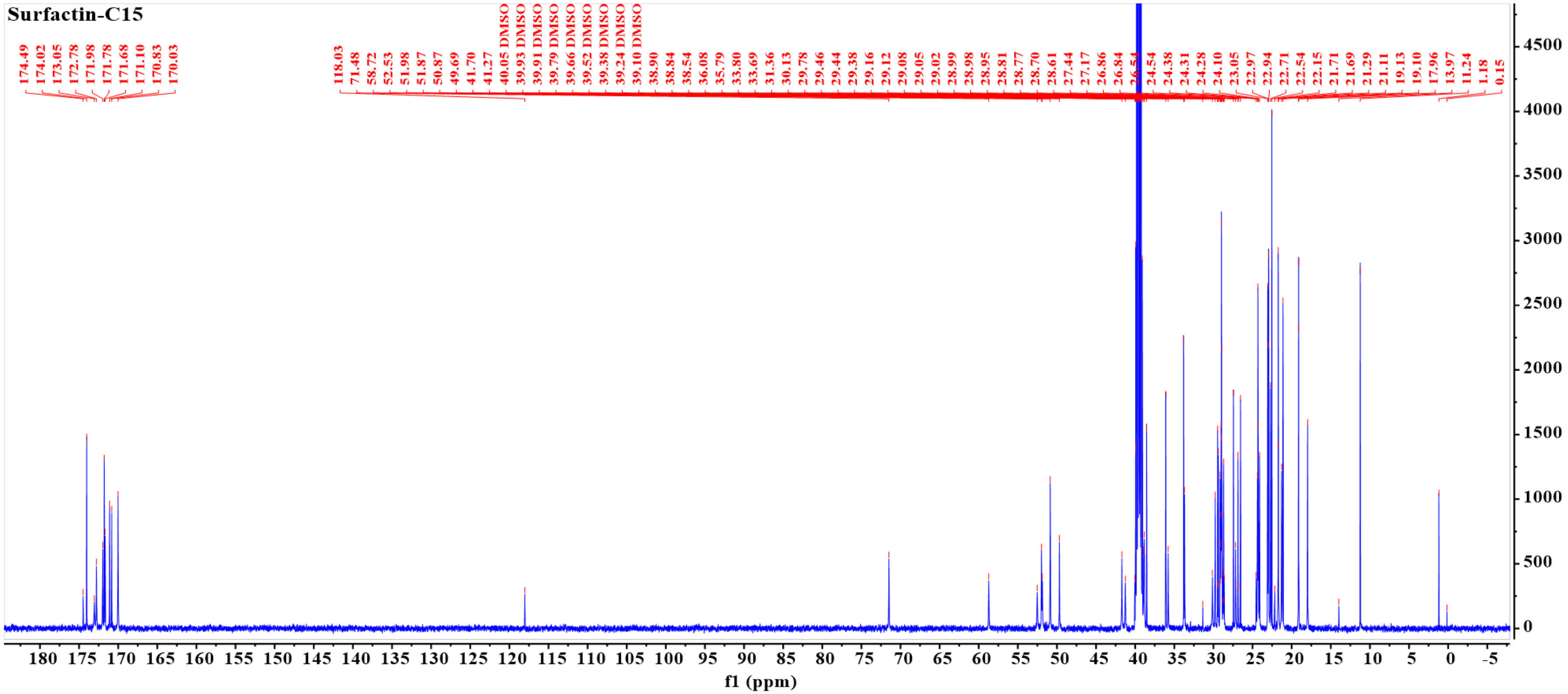

Supplementary Figure 7. ^{1}H-NMR (a) and ^{13}C-NMR (b) spectra of component 4 (surfactin-C15).

Supplementary Table 2. MS/MS ion assignments of surfactins generated from the [M +Na]$^+$ ion peaks (y8 and b8)

Compound		Ion type						
	**y8**	**y7**	**y6**	**y5**	**y4**	**y3**	**y2**	**y1**
2	1030	836	707	594	481	382	267	/
3	1044	/	707	594	481	382	267	/
4	1058	/	707	594	481	382	267	/
	**b8**	**b7**	**b6**	**b5**	**b4**	**b3**	**b2**	**b1**
2	1030	917	804	689	590	477	364	235
3	1044	931	818	703	604	/	/	/
4	1058	945	832	717	618	/	/	/

Supplementary Table 3 ^{13}C- and ^{1}H-NMR spectral data for three kinds of algicidal compounds in DMSO.

Amino acids	Position	Surfactin-C13		Surfactin-C14		Surfactin-C15	
		δc (ppm)	δ_H (ppm)	δc (ppm)	δ_H (ppm)	δc (ppm)	δ_H (ppm)
Glu_1	NH		7.85 (d,6.19)		7.83 (d,6.22)		7.83 (d,5.94)
	α-C	52.5	4.17 m	52.5	4.17 m	52.5	4.17 m
	β-C	27.2	1.93 (m)/1.80 (m)	27.2	1.93 (m)/1.80 (m)	27.2	1.93 (m)/1.80 (m)
	γ-C	29.8	2.25 (m)	29.8	2.25 (m)	29.8	2.25 (m)
	CO	171.1		171.1		171.1	
	δ-CO	174.0		174.0		174.0	
Leu_2	NH		7.99 (s)		7.99 (s)		8.0 (s)
	α-C	52.0	4.17 (m)	52.0	4.17 m	52.0	4.17 (m)
	β-C	39.5	1.53 (m)	39.5	1.53 (m)	39.5	1.53 (m)
	γ-C	24.4	1.53 (m)	24.3	1.53 (m)	24.4	1.53 (m)
	δ_1-C	23.0	0.85 (m)	23.0	0.85 (m)	23.0	0.85 (m)
	δ_2-C	23.1	0.85 (m)	23.1	0.85 (m)	23.1	0.85 (m)
	CO	173.0		172.9		173.0	
Leu_3	NH		8.13 (d,7.35)		8.13 (d, 7.31)		8.12 (d,7.1)
	α-C	52.0	4.17 (m)	52.0	4.17 m	52.0	4.17 (m)
	β-C	39.0	1.52 (m)	39.0	1.52 (m)	39.1	1.52 (m)
	γ-C	24.4	1.52 (m)	24.3	1.52 (m)	24.4	1.52 (m)
	δ_1-C	22.7	0.85 (m)	22.7	0.85 (m)	22.7	0.85 (m)
	δ_2-C	23.0	0.85 (m)	23.0	0.85 (m)	23.0	0.85 (m)
	CO	172.9		172.7		172.8	
Val_4	NH		8.03 (s)		8.03 (s)		8.06 (s)
	α-C	58.7	4.04 (t,7.5)	58.7	4.04 (t,7.8)	58.7	4.03(t,7.5)
	β-C	30.2	2.01 (m)	30.1	2.01 (m)	30.1	2.01 (m)
	γ_1-C	18.0	0.75(m)	18.0	0.75 (m)	18.0	0.75 (m)
	γ_2-C	19.2	0.85 (m)	19.1	0.85 (m)	19.1	0.85 (m)
	CO	171.1		170.8		170.8	
Asp_5	NH		8.14 (d,7.35)		8.14 (d, 7.31)		8.13 (d,7.1)
	α-C	49.7	4.55 (m)	49.7	4.55 (m)	49.7	4.56 m
	β-C	35.8	2.73(dd,4.84,16.71)/ 2.59 (dd,9.39,16.63)	35.8	2.73 (dd,4.84,16.69) 2.59(dd,9.32,16.75)	35.8	2.74 (dd,4.67,16.72)/ 2.60 (dd,9.35,16.68)
	δ-CO	172.0		171.7		171.7	
	CO	170.0		170.0		170.0	
Leu_6	NH		7.62 (d,8.43)		7.62 (d,8.24)		7.61 (d,8.37)
	α-C	50.9	4.36 (m)	50.9	4.36 (m)	50.9	4.36 (m)
	β-C	41.7	1.49 (m)	41.7	1.49 (m)	41.7	1.49 (m)
	γ-C	24.3	1.49 (m)	24.3	1.49 (m)	24.3	1.49 (m)
	δ_1-C	21.7	0.80 (m)	21.7	0.80 (m)	21.7	0.80 (m)
	δ2-C	22.6	0.80 (m)	22.7	0.80 (m)	22.6	0.80 (m)
	CO	171.8		171.6		171.6	
Leu_7	NH		8.43(d,7.54)		8.43(d,7.74)		8.43 (d,7.34)
	α-C	50.9	4.06 (m)	50.9	4.06 (m)	50.9	4.06 (m)
	β-C	39.0	1.49 (m)	38.9	1.49 (m)	38.9	1.49 (m)
	γ-C	24.1	1.49 (m)	24.2	1.49 (m)	24.2	1.49 (m)

	δ_1-C	21.1	0.85 (m)	21.1	0.85 (m)	21.1	0.85 (m)
	δ2-C	21.2	0.85 (m)	21.3	0.85 (m)	21.3	0.85 (m)
	CO	172.8		172.0		171.9	
Fatty acid	1	170.0	—	170.0	—	170.0	—
	2	41.2	2.49 (m)/2.33 (m)	41.2	2.49 (m)/2.33 (m)	41.3	2.49 (m)/2.32 (m)
	3	71.5	5.07 (m)	71.5	5.07 (m)	71.5	5.08 (m)
	4	33.8	1.52 (m)	33.8	1.52 (m)	33.8	1.52 (m)
	5	24.4	1.23 (m)	24.3	1.23 (m)	24.4	1.23 (m)
	6-8	28.6-29.2	1.23 (m)	28.6-29.4	1.23 (m)	28.6-29.4	1.23 (m)
	9	36.1	1.11 (m)/1.31 (m)	28.6-29.4	1.23 (m)	28.6-29.4	1.23 (m)
	10	33.8	1.23 (m)	26.8	1.23 (m)	28.6-29.4	1.23 (m)
	11	26.4	1.25 (m)	38.5	1.11 (m)/1.31 (m)	36.1	1.11 (m)/1.31 (m)
	12	11.3	0.85 (m)	27.5	1.25 (m)	33.8	1.25 (m)
	13	19.1	0.85 (m)	22.6	0.85 (m)	26.5	1.25 (m)
	14			22.6	0.85 (m)	11.2	0.85 (m)
	15					19.1	0.85 (m)
